# Common Clinical Trial Amendments, why they are submitted and how they can be avoided: a mixed methods study on NHS UK Sponsored Research (Amendments Assemble)

**DOI:** 10.1186/s13063-022-06989-0

**Published:** 2023-01-04

**Authors:** Shivam Joshi

**Affiliations:** University Hospitals Coventry & Warwickshire NHS Trust, Research & Development, Clifford Bridge Road, Coventry, CV2 2DX UK

**Keywords:** Amendment, Clinical trial, Research waste, Feasibility, Protocol, NHS

## Abstract

**Background:**

Amendments are changes made to a clinical trial after it has received regulatory approval. An amendment can take a significant amount of time and resources to develop, review and implement at participating sites. This can affect the efficient delivery of clinical trials and potentially contribute to research waste. This study aimed to establish what the most common amendments are, why they are submitted, and what, if anything, can be done to avoid them.

**Methods:**

An explanatory sequential mixed methods design was employed. The first strand involved a content analysis on a sample of amendments, submitted in trials sponsored by a University Hospital NHS Trust between September 2009 and March 2020, to establish the most common changes and reasons for amendments. The second strand involved thematically analysing semi-structured interviews with trial stakeholders to explore their views on the reasons underpinning the submission of amendments, and the potential for efficiencies that could prevent avoidable amendments.

**Results:**

Two hundred forty-two approved amendments were examined from 53 clinical research studies. The ‘Addition of sites’ was the most common amendment change, and the most common reason for amendments was ‘To achieve the trial’s recruitment target’. The root causes for avoidable amendments identified by the 11 interviewees included the following: ‘Rushing the initial application knowing an amendment will be needed later’, ‘Not involving all the right people to input’ at the start of the trial, and ‘Realising it’s not feasible in practice when delivering the trial’. Missing regulatory checks following an onerous and error-prone application process were also identified as the cause of some amendments.

**Conclusions:**

Trials need to be critically reviewed by various stakeholders and have sufficient time allocated to planning and feasibility assessments to avoid some amendments. This may improve clinical trial efficiency, to benefit the trial participants, researchers, funders, sponsors, and regulatory bodies, and potentially bring new treatments to patients faster.

## Background

All clinical trials must be conducted in compliance with the approved protocol [1, 2]. However, sometimes changes need to be made to the protocol or other trial documents after they have received regulatory approval. These changes are called amendments. An amendment can be substantial or non-substantial depending on the type of change being made. By definition ‘A substantial amendment is a change that is likely to have a significant impact on the safety or physical or mental integrity of the clinical trial subjects, or the scientific value of the clinical trial’ [1].

A Cochrane systematic review of studies comparing a trial’s initial plan with their final report showed that amendments made to trials are common, but underreported [3]. Two studies on multinational commercial trials by Getz et al. showed that 57% and 58.8% had submitted at least one amendment [4, 5]. These trials achieved lower recruitment than initially planned, compared to trials with no amendments [4]. Getz et al. revealed the most common amendment was a change to the trial population description, which included changes in eligibility criteria [5]. While the most common reason for an amendment was due to the availability of new safety information [5]. Pressure to collect more data and recruit to target were also identified as underlying problems that lead to amendments [5].

Getz et al. suggest that between one third and 45% of amendments could have been avoided [4, 5], citing flaws in trial design, inconsistencies in the protocol, unfeasible eligibility criteria and recruitment challenges as causative factors [4, 5]. However, they only considered substantial protocol amendments in commercial trials. Therefore, their findings may be less applicable to non-commercial sponsors, such as the NHS, where there is currently limited research on clinical trial amendments.

There are various types of clinical research carried out in the NHS, and for the purposes of this study, they will be referred to collectively as clinical trials. To request a clinical trial amendment in the UK, an amendment form needs to be submitted to a regulatory body. One form may include multiple amendments, summarising the changes made and justification for them. The form may also refer to other supporting text, along with any trial documents being amended, with changes clearly marked [6].

Regulatory approval is required before an amendment can be implemented [1]. Regulatory bodies (such as the Research Ethics Committee (REC) [7], Medicines and Healthcare products Regulatory Agency (MHRA) [1] and most recently introduced body the Health Research Authority (HRA) [7]) required to review the amendment will depend on the type of clinical trial and amendment being made. An amendment can then be approved or rejected. Those that are rejected cannot be implemented but can be re-submitted as a modified amendment [6].

Amendments can be a significant burden on regulatory bodies. A Freedom of Information (FOI) request (FOI Ref: 2021/FOI/031) revealed that the HRA had processed a total of 18,309 amendments in England and Wales during April 2019–March 2020; of these, 58% were substantial amendments. The MHRA receive approximately 5500 substantial amendments to review each year [8]. The time taken to receive approval for an amendment can disrupt the running of a clinical trial. RECs usually give approval within 35 calendar days of receiving a valid amendment [1, 6]. However, during April 2019–March 2020, the average number of days to HRA approval following the submission of a substantial amendment was 48 days, and 1 day for non-substantial amendments (FOI).

Each amendment takes a significant amount of time to be developed by the Chief Investigator (CI) and their team, then be reviewed by the sponsor, regulatory bodies, and sites, before being implemented. The amendment process can take up administrative and clinical resource time, as well as funding (substantial amendments made to the MHRA cost £225 per amendment [9]). Getz et al. reviewed 21 phase III protocol amendments and found them to have a median direct cost of $535,000 (USD) to implement [4]. The actual costs are likely to be much higher if including indirect costs such as staff time. This resource time and funding could be better spent on improving data quality and recruitment of trial participants.

Amendments can thus directly affect the efficient delivery of clinical trials and potentially contribute to research waste. While some amendments may be unavoidable, some can be avoided by consideration at the early trial development stage before obtaining regulatory approval. This study aimed to find out what the most common amendments are, why they are made and how clinical trial efficiency could be improved by reducing them.

## Methods

This study employed an explanatory sequential mixed methods design [10]. The first strand involved the content analysis of a sample of amendments submitted by University Hospitals Coventry & Warwickshire (UHCW) NHS Trust. This was followed by the second strand which involved conducting semi-structured interviews with trial stakeholders working at UHCW NHS Trust. The findings from the content analysis were presented to the interviewees to gather their insights and to provide more depth of understanding to the first strand [10].

### Content analysis

A conventional content analysis approach was employed which derives categories directly from the text data [11], using the inductive content analysis steps described by Elo and Kyngäs [12].

Only electronically accessible amendments for clinical trials solely sponsored by UHCW NHS Trust and submitted before 16th March 2020 were included in this study. This date was chosen as a natural cut-off following the suspension of many trials after the COVID-19 pandemic lockdown measures [13].

Each eligible trial was given a unique ID number, and any amendments were sequentially numbered and recorded on an Excel database. Where an amendment was submitted to modify an existing amendment (*n* = 1), these amendments were reviewed together and merged as a single amendment for inclusion in this study. Where the regulatory body requested an amendment to be resubmitted as substantial rather than a non-substantial amendment, or vice versa, then only the approved amendment was included (*n* = 2), as the content for these amendments were the same.

The amendment form for each trial amendment was used as the primary source of text data. However, if the amendment form was not available in the trial’s electronic folder or further information was required to understand the changes made, then other documents detailing the amendment changes were reviewed. For example, protocols, cover letters, and other correspondence.

Individual amendment ‘Changes’ and ‘Reasons’ were chosen as the recording units. The amendment text data were read in full and inductively coded for changes made and reasons for the amendment where stated. If a specific amendment change or reason was repeated in the same amendment text, it was coded together in a single code, to prevent double counting [14].

The first amendment coded by the researcher was independently reviewed by an experienced mixed methods researcher. After coding each amendment, 5% of the sample of amendments (*n* = 12) were randomly selected using an online Random Sequence Generator to be independently coded by a second coder. The content analysis proceeded after this reproducibility was confirmed [14].

NVivo 12 Plus was used to group the amendment codes into content-related categories, under the broader category headings ‘Changes’ and ‘Reasons’. Each category was reviewed by the researcher to ensure the codes fitted within the category’s definition, and if not, the code was correctly re-assigned. This checking also ensured there were no duplicate categories. The final list of ‘Changes’ and ‘Reasons’ categories were exported. The number of node references for each individual category indicated the count frequency of each category’s occurrence in the sample.

### Interviews

Around 150 staff at UHCW NHS Trust involved in research as part of their role were invited by email to take part in a research interview, to explore their views and experiences of clinical trial amendments. Further information was explained in an information sheet. To be eligible, staff had to have experience of either the development, review, or implementation of at least three clinical trial amendments. Those that expressed an interest to take part were asked to electronically complete and return a consent form and data collection form.

The interviews were semi-structured [15] and conducted via Microsoft Teams or telephone by the same researcher. Interviewees were asked about the most common changes and reasons for amendments from their experience, before being shown the findings of the content analysis to comment on. Interviewees were also asked about their views on how amendments could be avoided and further probed on why these activities do not happen in practice.

Interviews were captured on a PC Voice Recorder App and the Otter.ai online recorder which supports auto-transcription. To avoid potential information bias, at the end of each interview the interviewees were reminded not to discuss the contents of the interview with colleagues until all the interviews had been completed.

All interviews were listened to again by the researcher, who made any edits required to the transcripts to make sure all interviews were transcribed verbatim. Transcripts from each interview were uploaded to NVivo 12 Plus for analysis and analysed thematically using the Framework approach [15].

Each interview transcript was coded by the researcher into broad categories based on areas of discussion in the topic guide. Following the initial coding of a few interview transcripts, feedback was sought from an experienced mixed methods researcher, to refine coding and ensure that meanings were not lost. Grouped codes were reviewed to confirm data extracts aligned to the theme description.

Connections between themes were reviewed and re-organised to find patterns in the data, and to further refine the themes. This was supported by creating Framework matrices in NVivo to compare themes both within and across cases, using the original coded text as summaries. To help map the links between the data, some of the key themes and sub-themes were printed out and re-grouped during this abstraction and interpretation stage [15]. Methods triangulation was also carried out to compare the interview data with the content analysis findings [15].

## Results

### Content analysis

Fifty-three percent of trials submitted at least one amendment (Table 1), with a mean of 4.5 amendments per trial. All four Clinical Trials of Investigational Medicinal Products (CTIMPs) in the sample submitted at least one amendment. The largest total number of amendments submitted by a single trial was 34, a non-commercially funded Randomised Control Trial (RCT) CTIMP.

**Table 1:** Summary of trial characteristics further split by those with and without an amendment submitted

	Trials with amendments submitted (*n* = 53) *n*(%)	Trials with no amendments submitted (*n* = 47) *n*(%)	All included trials (*n* = 100) n(%)
Trial status			
Open to recruitment^a^	32(60)	21(40)	53(53)
Closed to recruitment	21(47)	24(53)	45(45)
Not started recruitment	0(0)	2(100)	2(2)
Trial design			
Observational	37(51)	36(49)	73(73)
Non-randomised intervention	2(40)	3(60)	5(5)
Randomised control trial (RCT)^b^	14(64)	8(36)	22(22)
Trial funder			
Commercial	8(89)	1(11)	9(9)
Non-commercial	28(82)	6(18)	34(34)
Not funded	10(34)	19(66)	29(29)
Not known	7(25)	21(75)	28(28)

^a^This includes trials where it was not clear if the study had closed, so was assumed to still be open to recruitment; it also includes those trials subsequently known to have paused recruitment due to the COVID-19 pandemic

^b^A total of 4 are CTIMPs, and all 4 had amendments submitted

**Fig. 1:**
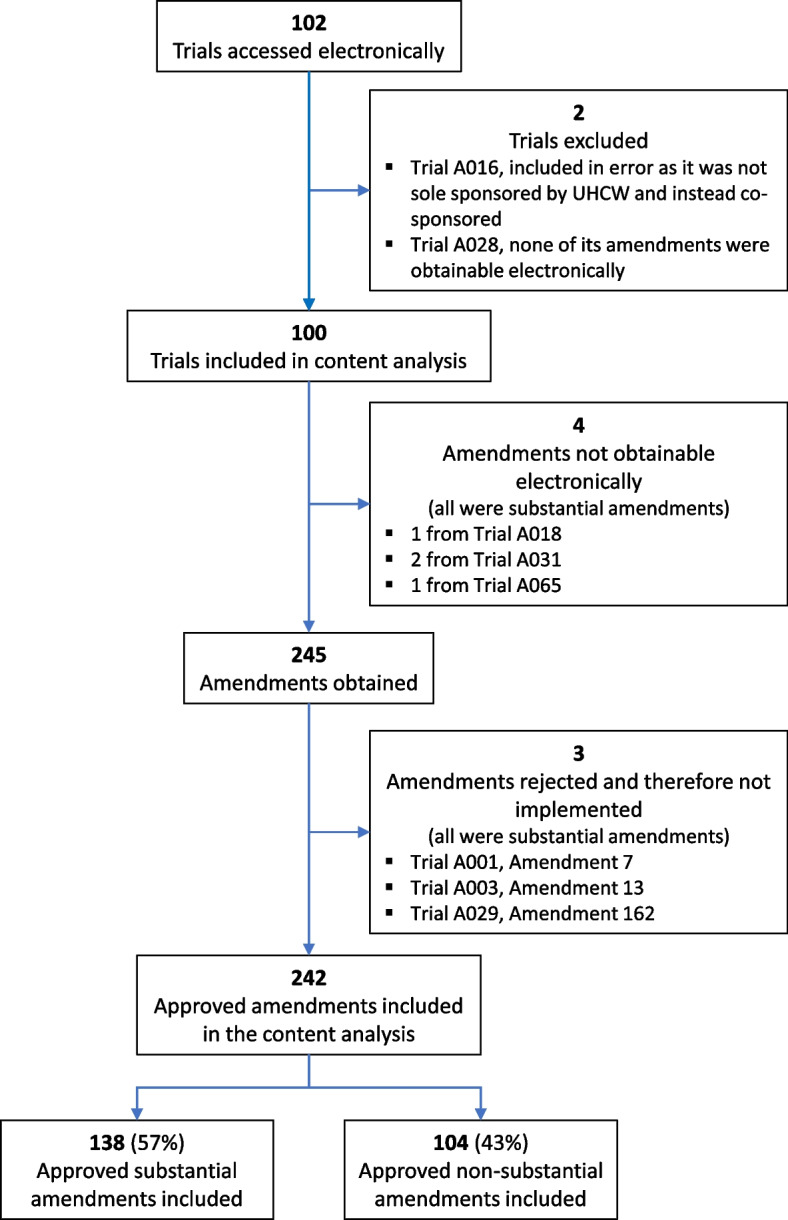
Summary of trials and amendments obtained and included in the content analysis

Two hundred forty-two regulatory body-approved amendments were included in the content analysis (Fig. 1), 57% of which were substantial amendments. These amendments were submitted between 24 September 2009 and 10 March 2020.

**Fig. 2:**
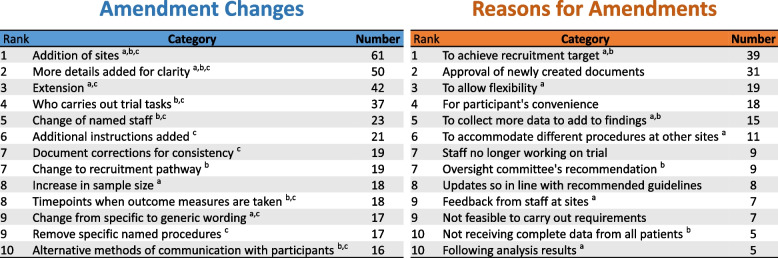
Content analysis findings displaying the most common content categories for amendment changes and reasons for amendments. N.B. Content categories that had the same frequency count were ranked the same. This visual also integrates data gathered from the interviews; the content categories are annotated with feedback from the interviewees after viewing these findings, where some interviewees had stated that **a** they commonly see these categories, **b** they see less of these categories and **c** these categories could have been avoided

The content analysis produced 1229 amendment codes. Nine hundred twenty-six codes were referenced under 168 categories for ‘Amendment Changes’, and 303 codes referenced under 81 ‘Reasons for Amendments’ categories. Figure 2 summarises the top 10 most frequently referenced content categories. The most common amendment change in the sample were amendments to request the addition of sites (*n* = 61). The most common reason for an amendment was to achieve the trial’s recruitment target (*n* = 39).

### Interviews

Eleven members of UHCW NHS Trust staff volunteered to take part in the interviews over a fortnight in September 2020. The interviews were remotely conducted via video call (*n* = 8) or phone call (*n* = 3) and lasted between 21 and 67 min (44 min mean duration). Seven female and four male staff took part, with a mean age of 41 years. Table 2 summarises the other interviewee’s characteristics.

**Table 2:** Summary of interviewee characteristics

Interviewee ID	Workforce category	Job role area
A01	Clinical	Researcher
A02	Non-clinical	Researcher
A03	Clinical	Clinical delivery staff
A04	Clinical	Researcher
A05	Clinical	Clinical delivery staff
A06	Non-clinical	Research support staff
A07	Non-clinical	Research support staff
A08	Non-clinical	Research support staff
A09	Non-clinical	Research support staff
A10	Clinical	Clinical delivery staff
A11	Clinical	Researcher

The ‘Workforce category’ refers to whether the interviewee’s job role is ‘Non-clinical’ typically not patient-facing or ‘Clinical’ typically patient-facing which included two consultants and a research nurse. The ‘Job role area’ was assigned by the researcher to group the interviewees into broad categories. ‘Researcher’ were those who led their own research as part of their role, e.g. Chief Investigator; ‘Clinical delivery staff’ were Research & Development (R&D) staff involved in clinical aspects of delivering trials, e.g. Research Nurse; and ‘Research support staff’ were non-clinical R&D staff involved in supporting the set-up or management of trials

Collectively, the group had 99 years of experience with clinical trial amendments. Interviewees were involved in amendments from either the sponsor or participating site perspective, or they had experience of both. Some interviewees were familiar with the trials included in the content analysis, while others had experience running their own research project, with five having completed a postgraduate qualification. Three of the interviewees had the experience of leading clinical trials as a CI and supervising junior researchers as part of their role. While all interviewees were employed by UHCW NHS Trust, some had experience working in the commercial sector, a Clinical Trials Unit, and in an academic setting.

The researcher who carried out the interviews also worked at UHCW NHS Trust. Applying the same interviewee characteristics in Table 2, the researcher’s role is non-clinical, in the research support staff category.

#### Amendment changes mentioned by interviewees

Before being shown the findings of the content analysis, interviewees were asked about the most common amendment changes made in their experience. Table 3 lists all the amendment changes mentioned.

**Table 3:** Amendment changes mentioned by interviewees along with the number that had mentioned each

Amendment change	Number of interviewees mentioning this amendment change
Addition of sites	4
Change of PI	4
Change to eligibility criteria^a^	4
Change to PIS wording	4
Change to protocol	3
Extension of study	3
Sub-studies (inc. COVID)^a^	3
Typo changes	3
Change to advertisement material	2
Changes to Reference Safety Information (RSI) safety data^a^	2
Increase in sample size	2
Related to trial drug^a^	2
Adding additional data	1
Adding clarifications	1
Adding extra visits	1
Addition of arms in drug trials^a^	1
Additional procedures^a^	1
Additional safety procedures^a^	1
Additional visits^a^	1
Allow contact of patient prior to appointment	1
Change from specific to generic wording	1
Change of post-dose observational follow-up time^a^	1
Change of questionnaire wording	1
Change to data collection tools^a^	1
Change to design and trial flow	1
Change to patient communication methods (inc. due to COVID)	1
Change to statistical analysis plans^a^	1
Changes to intervention^a^	1
Changes to recruitment process	1
Changing Serious Adverse Events (SAE) process and definitions	1
Collecting additional samples	1
End of recruitment date change	1
Making wording more suitable for laypeople	1
Taking off hold (inc. due to COVID)	1

^a^Amendments that some interviewees were surprised did not appear in the content analysis findings presented (Fig. 2)

The ‘Addition of sites’ was one of the most common amendments mentioned by interviewees, with one suggesting that they are inevitably part of the trial journey.

it’s very rare that you can get through a big multicentre study without adding sites...I think addition of sites is something that will happen. [A11]

Extensions were mentioned by three interviewees but were seen as an unfavourable amendment by some. The frustration it caused to site staff was raised, further impacting trial delivery, as well as the scientific integrity of data being compared over a longer period.

you’re in at a different place completely as to when your first patient went in four years ago. So, you know, the comparisons are less robust. [A03]

#### Interviewee’s feedback on the content analysis findings

Seven interviewees said they were not surprised by the content analysis findings, as shown in Fig. 2. Feedback from interviewees has been annotated in Fig. 2. Specifically in relation to which categories they commonly saw, ones they saw less of, and categories which they thought could have been avoided.

The highest ranked content categories, the ‘Addition of sites’ and ‘To achieve recruitment target’, were both mentioned by interviewees as commonly seen, but also seen less often by some. This highlighted that some interviewees spent less time focussing on certain amendments as part of their role. For example, staff working in pharmacy spent less time reviewing amendments unrelated to trial drugs.

Some amendments were suggested to be more likely submitted by a commercial sponsor compared to a non-commercial one, such as the addition of sites and changing of drugs during a trial. Less restricted funding was mentioned as a reason for these amendments being more common in commercially sponsored trials.

Amendments which interviewees were surprised did not appear in the content analysis findings are indicated with an asterisk in Table 3. This included the ‘Change to eligibility criteria’ which was mentioned by four interviewees. Methods triangulation later revealed that the amendments mentioned by interviewees (Table 3) could all be attributed to the full list of ‘Amendment changes’ content categories. This supports the overall validation of the data [15, 16].

A ‘Change to recruitment pathway’ and an ‘Increase in sample size’ were both not mentioned as avoidable amendments. One interviewee was also surprised at how frequently an ‘Increase in sample size’ appeared, emphasising that this should only be done if scientifically justifiable to ensure good research practice.

#### Reasons for amendments

**Fig. 3:**
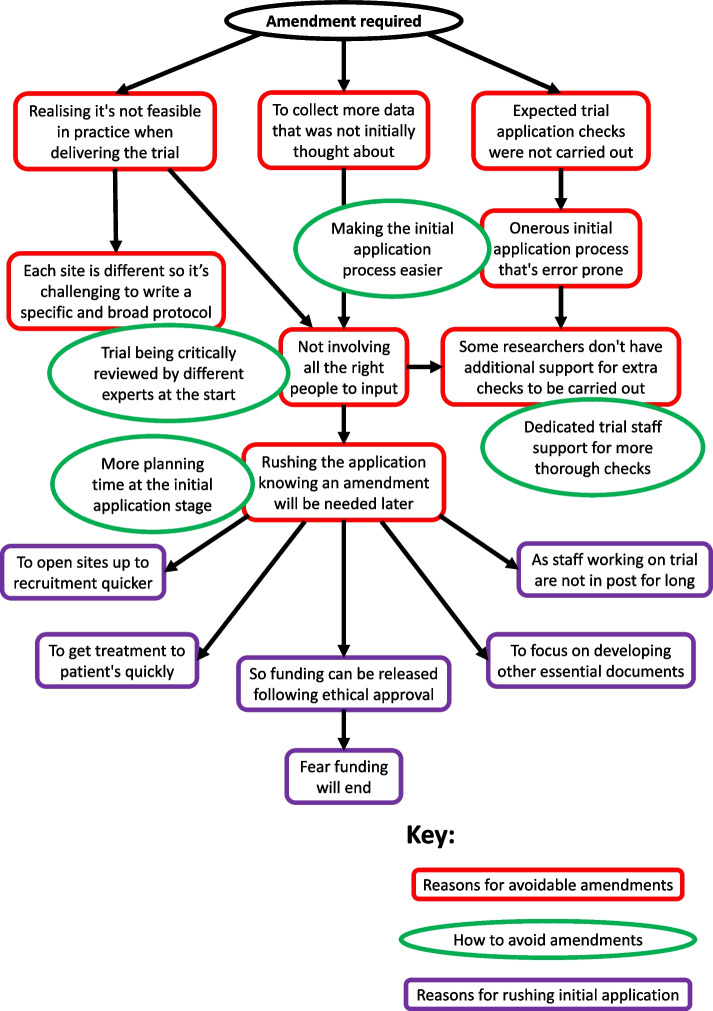
Main themes identified from the interviews to find the root cause for why amendments are made and how they can be avoided

The reasons provided by interviewees focused on oversights made at the initial application stage, which led to potentially avoidable amendments being made. The primary reasons that cause avoidable amendments are summarised into three themes and presented in red boxes at the top of Fig. 3. These are ‘Realising it's not feasible in practice when delivering the trial’, ‘To collect more data that was not initially thought about’ and ‘Expected trial application checks were not carried out’. These will be further explored below along with the other connected themes, to reveal the root cause of amendments. Themes identified for how to avoid amendments are presented as green circles overlapping the themes they are linked to.

##### Realising it's not feasible in practice when delivering the trial

Eight interviewees stated that, after a trial is open to recruitment, mistakes are realised that make it unfeasible to deliver, and therefore, amendments are made to rectify them. This was mainly put down to unrealistic patient and site feasibility assessments carried out by sponsors.

I think there are some, there’s some gaps in in feasibility as to exactly what can…happen, what can be done…have you got the staff have you got the equipment. Have you got the time, have you got the expertise so you’ve got the knowledge and you’ve got the training. [A03]

Lack of feasibility also encompassed the need for amending the trial to achieve the recruitment target. Examples provided included amending eligibility criteria which restricted their recruitment and making the trial procedures more convenient for participants, to improve recruitment and retention to the trial. As new sites are opened the trial becomes open to more scrutiny and review, with changes required to accommodate them as well as their patients.

I’ve had studies where we’ve recommended amendments to the patient information sheet because the nurses have said, this doesn’t seem to be working or, I don’t think this is explained in enough detail. [A07]

One of the challenges identified to site feasibility was that ‘Each site is different so it’s challenging to write a specific and broad protocol’, which incorporates each site’s standards of care and pathways.

So it is quite a fine art writing a protocol to make it specific enough that we need that it’s doing what we need it to do. And we’re recruiting and identifying or, you know, patients are following the same pathway, but it needs to be broad enough that every site can apply it to their local setting. And I think that’s that’s quite a challenge. [A06]

With their experiences of multi-centre trials, interviewees grouped in the ‘Researcher’ job role did not mention site differences as a challenge, when comparing this theme across interviewees.

##### To collect more data that was not initially thought about

Six interviewees mentioned that the expansion of clinical trials to collect more data was a common reason for submitting amendments. It was suggested that researchers may find interesting data during the trial that they want to explore further. This included adding sample collections, sub-studies, and follow-up visits. Concerns were raised about this practice, with its impact on participant recruitment and retention, and time taken to deliver the trial.

And, you know, maybe everything that they asked for has to be credible and scientifically appropriate for the scientific question of the protocol and not have things bolted on. Just because they might be interesting. [A03]

Researchers wanting to collect additional data and the lack of feasibility during a trial were both associated with ‘Not involving all the right people to input’ at the trial development stage.

I think it’s about having the right people, not just having every…anybody going oh yeah I’ll have a look at it oh get so and so to have a look at it and I think it having the right people involved, especially your clinical team I think makes a big impact on the type and the quality of your project in the beginning. [A08]

It was suggested that researchers tend to only involve those they know, potentially leading to errors requiring an amendment. However, there seemed to be a lack of this involvement even when researchers had access to clinical teams or other peers to review their documents. One of the reasons for this was put down to some researchers being protective to retain ownership of their trial.

A ‘Trial being critically reviewed by different experts at the start’ was suggested by eight interviewees to improve its quality and avoid amendments. The importance of having a multidisciplinary team of experts, including staff from different sites, was emphasised to understand their local practices. Five interviewees also recommended early and broad engagement with Patient and Public Involvement (PPI).

So, what I tend to do now is you know get the relevant stakeholders involved early if possible, and get input from as many people who are involved in the study in the beginning, and get the experiences shared experiences to know some of the challenges that you might face during the study, and I think you know that can minimise the, not completely negate but minimise the risk of amendments I think. [A01]

When asked why activities such as involving the right people at the start did not happen, five interviewees put this down to ‘Rushing the application knowing an amendment will be needed later’. This was seen as one of the main root causes for amendments in most situations. Therefore, the themes summarising the reasons for rushing are presented in purple boxes at the bottom of Fig. 3.

the [study name] trial is just just looking to open and they are pushing that through so quickly that my fear is that we’re going to end up with loads of amendments because it's been rushed. [A06]

Rushing to release grant money upon achieving ethical approval and the rush to open sites to recruitment appeared frequently, along with the fear of potentially losing funding as mentioned by one of the interviewees.

if you got ethics approval, it helps your site setup process. The big the big delay is site setup, in trials…it’s site review and setup. And no one really does anything until you’ve got ethics. [A11]

These reasons for rushing were seen as potential barriers to avoiding amendments. However, the ability to make amendments was seen as a positive action for some. One interviewee mentioned intentionally submitting the trial before their oversight committee had reviewed it, to obtain ethical approval, and then put forward any oversight committee recommendations as an amendment. They later advised that this order of activity should only be done where researchers already benefit from broad oversight, knowing that thorough checks will be done. Involving more people was also suggested to make the trial development process longer, which could eat into time allocated for recruitment, potentially leading to amendments to extend.

Seven interviewees had instinctively suggested that ‘More planning time at the initial application stage’ would help avoid amendments later.

So the planning is the planning and the feasibility is critical to the deliverability of a study. And if you get that right up front, then you should need less amendment and the trial should run much more smoothly. [A06]

Trial recruitment was suggested to be improved by planning which sites to engage with and selecting them on past performances.

maybe working on site intelligence, and performance of previous sites, maybe if the data is out there looking at sites that have performed well before for other studies, maybe with other companies. [A03]

To avoid amendments and trial limitations, one interviewee mentioned spending more time at the start considering what data can be collected.

So I would say that that’s one area that does get a lot of time and attention spent on it so that you can make sure that right from the start, you’re able to collect all the data from patients that you need. You know thing things like long term follow up data would be something that you need to make sure that it’s in the patient information leaflet and then the consent form. If there’s just a, you know, NHS digital long term yearly follow up or something like that. [A04]

##### Expected trial application checks were not carried out

Some interviewees expected certain checks to be carried out by sponsors and regulatory bodies at the initial application stage. Amendments were then required due to these missing checks. A few put this down to the composition of the REC not always having the relevant expertise for certain trials, while some assumed the introduction of HRA reviews would lead to fewer amendments. For example, when an interviewee raised issues with document inconsistencies requiring amendments, they said:

I think that that’s always been a concern and I think I thought when the HRA would come in that things like that would wouldn’t happen anymore. That it would be a lot more transparent, or the documents would be a lot clearer. [A07]

There was also an expectation the HRA would check if documents required for pharmacy review were submitted. Early site reviews of the pharmacy manual, which is not required for regulatory review, was recommended to prevent some amendments.

There are too many grey lines. I do think that the MHRA, REC et cetera could have been much clearer guide as to exactly where our responsibilities lie…And I feel that there needs to be further clarity on, from both a sponsor perspective and a regulatory body perspective on who needs informing of what and what is expected of sites. How thorough a review and et cetera should be made. [A05]

Mistakes in clinical trial documents were also put down to an ‘Onerous initial application process that's error prone’. This encouraged activities during trial development which could lead to document inconsistencies. Such as, cutting and pasting text from other trials and between documents of the same trial due to duplication between the protocol and the Integrated Research Application System (IRAS).

there’s also a lot of a lot of repetition between documentation, in the application, which also leads to faults and mistakes that you shouldn't need to do. [A02]

Amendments were also seen as an easier alternative to the time-consuming initial application process.

the good thing about amendments is they’re quite short, they’re actually quite short from an administration point of view, much easier than the main form. [A11]

Therefore, interviewees suggested amendments could be avoided by ‘Making the initial application process easier’. With less duplication between documents and agreeing standard templates for guidance and consistency.

Another prominent reason for amendments was that ‘Some researchers don’t have additional support for extra checks to be carried out’. This included a team to review their initial application. Staff support was suggested to influence both the number and quality of amendments submitted. However, an interviewee also mentioned that it becomes harder to gain support from experts with the most experience as they themselves are busy.

it does come down to time commitment, and getting because the more you want to get in terms of experience and stakeholders, but then more difficult it becomes to organise a meeting … that’s where the problems lie unfortunately the more experience you want to garnish and gather…you know the more people you have to try and get to a certain specific timeline. [A01]

It was consequently suggested there should be ‘Dedicated trial staff support for more thorough checks’ at the start, and to coordinate any feedback received. Five interviewees also mentioned personal experience helped them to better develop trials and avoid amendments. Table 4 summarises the interviewees’ suggestions on how to avoid specific amendments.

**Table 4:** Themes for avoiding specific amendments that were mentioned by interviewees

How to avoid amendments
Addition of sites should have another way to add these as a ‘notification’ instead, e.g. stating the approximate number of sites to be added instead and informing the regulatory body of new sites added as part of annual progress reports, without amending the original application form^a^
Allow whatever works locally at sites
Allowing flexible consent statement changes to version and date without an amendment
Being less specific and more flexible and inclusive where possible
Being specific about definitions and what sites can do
Casting the net wider for site recruitment
Flexibility of drug brands that can be used where possible
Having communication flexibility from the beginning where ethical
Include extended date from outset
Make visits follow standard care practice
Not including unnecessary eligibility criteria age limits
Only include procedures required for analysis
Planning numerous different potential recruitment pathways
Setting realistic site recruitment goals based on site feasibility and patient population
Using generic procedure names
Writing staff roles instead of specific names

^a^It was mentioned that this approach would not be applicable to CTIMPs

Eight interviewees also acknowledged that amendments are not completely avoidable, for example, when required for unpredictable patient safety reasons. Therefore, amendments were also seen as an inevitable part of research to resolve unexpected challenges, even when a wide range of experts are involved.

So I think the first point is wouldn’t necessarily see amendments as a bad thing I think it’s part of kind of the evolution of the trial design and. And as I say they can be quite useful because in a way, knowing that you can make amendments. Because, for unexpected challenges but you know there’s always going to be a challenge, it’s quite small, actually, because you can never get it perfect first time around. It’s just too big, they’re just too big and complex to get perfect. [A11]

## Discussion

Amendments can affect the efficient delivery of clinical trials. It is therefore important to find out what the most common amendments are and why they are made. This study found the ‘Addition of sites’ to be the most frequent amendment submitted in clinical trials sponsored by an NHS Trust. This amendment was also frequently mentioned by some interviewed trial stakeholders but seen less often by others if irrelevant to their role. In contrast, commercially sponsored trials most frequently amend their trial population description, including changes to the eligibility criteria [5]. Some interviewees in this study also mentioned a change to the eligibility criteria as a common amendment and were surprised it did not appear higher in the content analysis findings.

This study revealed the most frequent reason for an amendment was to achieve the trial’s recruitment target. Instead, Getz et al. found this to be the fifth most common reason [5], potentially highlighting some disparity between commercially and non-commercially sponsored trials. The reasons for amendments identified in the content analysis focused on why specific changes were made. These could be considered unavoidable at the time of submitting the amendment. This may be as amendment forms tend to focus on the present cause, such as an issue with recruitment, rather than potentially admitting a past oversight that led to the amendment. In contrast, these underlying reasons were readily revealed by the interviewees.

One of the primary root causes identified was rushing the initial application for regulatory approval to open sites quickly. However, connecting the findings of this study suggests potential feedback loops, which could theoretically lead to cycles of avoidable amendments. For example, rushing the initial application will mean less time is spent planning and involving relevant stakeholders, including site staff. This can subsequently result in poor site feasibility, especially as individual sites can work in different ways. Therefore, amendments may be required to accommodate a site or their patients. This can further delay the opening of sites to recruitment, which contradicts the rationale for rushing in the first place. Delays in recruitment may then lead to more sites being added in a rush and thus repeating the cycle. Amendments to extend a trial were also suggested to lead to site fatigue and a lack of motivation to recruit. This, combined with limited funding, can further affect a trial’s performance.

To break the cycle of avoidable amendments, more time needs to be spent planning and engaging with trial stakeholders early. Regulatory bodies also recommend this [1, 17] as well as early sponsor engagement with sites to confirm feasibility [18] prior to the submission of their application [7]. Some sites also prefer reviewing the protocol prior to submission and recommend a robust communications strategy to support this activity [19]. This may include formal site assessments and site selection visits [20].

The need for expert engagement is echoed when trying to reduce research waste more generally [21]. Ioannidis et al. recommend involving statisticians, methodologists and PPI from the outset, as well as training researchers on using appropriate methods, and rewarding those that produce reproducible research [21]. However, some interviewees found it difficult to obtain the expertise needed. The HRA’s ‘technical assurances’ review process [22] goes some way to help tackle this. Their review facilitates the identification and engagement of local experts and is intended to speed up site set-up activities [22]. Therefore, further support in this area is welcome.

The rigidity of protocols and need for flexibility was frequently highlighted by interviewees. Some experienced trialists may purposefully make their protocol less precise to avoid the need for an amendment [23]. Regulatory bodies already recommend accommodating local site variations from the outset, and including various text options in the Participant Information Sheet (PIS) for sites to choose from [6]. The COVID-19 pandemic further led the MHRA to encourage sponsors to add flexibility to trials. Using ‘hybrid designs’ to be able to adapt to changing rules both safely and reliably, whilst also avoiding amendments [24].

Regardless of ample regulatory advice, researchers continue rushing their initial application. Tackling some of the factors causing researchers to rush may alleviate any time pressure and allow them to spend more time planning. This should then save time and money associated with amendments. To achieve this, funders could actively encourage researchers to allocate more time to the planning phase of their grant applications. This may include time to co-produce their trial by engaging with stakeholders in the health service, academia, and the public [25]. Furthermore, including this activity as a milestone in funding contracts may reassure researchers that their awarded funding is secure. The additional planning time may erode into the intended recruitment phase, as suggested by some interviewees. Therefore, including a feasible recruitment timeframe in grant applications is also important. This may avoid initiating another feedback loop of amendments to extend a trial.

Some interviewees found it easier to submit an amendment compared to the initial application process. This implied a need to improve the application process and minimise duplication between trial documents. Comparably, amendments to correct trial documents for consistency were frequently seen in this study. The interviews also revealed the lack of clarity around who is responsible for checking what at the initial application stage. This could be a potential barrier to avoiding amendments, as simple document consistency checks can be missed. For example, the HRA indicate they will check to make sure the trial documents are consistent, are clear and accurately describe the research [26]. This expectation may lead to checks being missed by the sponsor and other reviewers. However, the HRA is responsible for assessing many applications, ensuring the risks to the participant, study and organisation are clearly outlined and mitigated where possible [26]. This may therefore lead to some errors being overlooked if they are deemed low risk. Research staff developing a trial should therefore be responsible for document consistency checks. To support this, regulatory bodies and sponsors should provide clear training to research staff on what they are responsible for checking.

## Strengths and limitations

One of the strengths of this study was its inclusion of all amendment types, not just substantial protocol amendments. Using a mixed methods approach also provided a rich understanding of non-commercially sponsored trial amendments.

As a single-centre study, the findings may not be transferable to other research organisations. In addition, the content analysis findings may have not revealed the full extent of amendments made during a trial, as 55% of included trials were not closed to recruitment.

Conventional content analysis can be criticised for missing categories related to the research question [11]. To address this, a random sample was second coded and the findings were triangulated with data collected from the interviews. Ideally, all the amendments would have been coded independently to improve reliability. Interviewees were also not asked to validate the findings, confirming if their words had been captured. This could have further improved the validity of the findings [15].

## Conclusions

Clinical trial amendments can take up a lot of time and resources which can be better spent on recruiting participants and improving data quality. Avoidable amendments can also contribute to research waste, as they impact on the efficient delivery of clinical trials.

This study found that the ‘Addition of sites’ was the most common amendment made and frequently experienced by trial stakeholders at UHCW NHS Trust. The most common reason for amendments was ‘To achieve recruitment target’. Instead, interviewees revealed various root causes for why potentially avoidable amendments are made, such as realising a lack of feasibility to deliver a trial in practice. To tackle this and avoid amendments, early planning and engagement with sites, stakeholders and a multidisciplinary team of experts is required. This should be encouraged by funders and considered as a milestone in funding contracts. Having dedicated staff to support these activities should be considered by sponsors, to ensure the initial application and trial documents are critically reviewed before submission. Furthermore, regulatory bodies and sponsors should provide clear training to research staff on their responsibilities, as well as the importance of planning and feasibility assessments to all trial stakeholders.

There is currently limited research carried out on clinical trial amendments. Therefore, the replication of this study by other non-commercial organisations is recommended, as gathering more transferable data in this field will further validate the findings. These can then be used to develop resources to prevent common avoidable amendments and improve clinical trial efficiency.

## Data Availability

The Clinical Trial Amendment data that support the findings of this study are available from UHCW NHS Trust, but restrictions apply to the availability of these data, which were used under licence for the current study, and so are not publicly available. All data generated or analysed during the current study are available from the corresponding author on reasonable request and with permission from UHCW NHS Trust.

## Abbreviations

CI: Chief Investigator
COVID: Coronavirus disease
CTIMPs: Clinical Trials of Investigational Medicinal Products
FOI: Freedom of Information
HRA: Health Research Authority
IRAS: Integrated Research Application System
MHRA: Medicines and Healthcare products Regulatory Agency
NHS: National Health Service
PI: Principal Investigator
PIS: Participant Information Sheet
PPI: Patient and Public Involvement
R&D: Research & Development
RCT: Randomised Control Trial
REC: Research Ethics Committee
RSI: Reference Safety Information
SAE: Serious Adverse Event
UHCW: University Hospitals Coventry & Warwickshire
UK: United Kingdom
